# Advising the immunocompromised traveller: a review of immunocompromise at The London Hospital for Tropical Diseases Travel Clinic between 1st April 2019 and 30th April 2020

**DOI:** 10.1186/s40794-024-00217-0

**Published:** 2024-04-15

**Authors:** Ellen Beer, Humayra Chowdhury, Bernadette Carroll, Akish Luintel, Christoffer van Tulleken, Nicky Longley

**Affiliations:** 1 University College London Hospital, London, NW1 2BU UK; 2 Hospital for Tropical Diseases, University College London Hospital, London, NW1 2BU UK; 3 London School of Hygiene & Tropical Medicine, Keppel St, London, WC1E 7HT UK

## Abstract

**Background:**

Immunocompromised travellers (ICTs) face greater infectious and non-infectious travel-associated risks than their immunocompetent counterparts. Increasing travel and emergence of novel immunosuppressants poses great challenges for travel medicine practitioners to confidently provide up-to-date evidence-based risk management advice and pre-travel care for ICTs.

**Methods:**

We reviewed the records of ICTs attending the London Hospital for Tropical Diseases (HTD) Travel Clinic between 1st April 2019 and 30th April 2020 with the aim to describe demographic and travel characteristics, type, and severity of immunocompromise, the degree of risk associated with intended travel and evaluate travel advice.

**Results:**

Of the 193 ICTs identified, immunocompromise was due to physiological reasons (42%), chronic infection (17.1%) and immunosuppressive therapy (16.6%). Median age was 38 (range 9 months to 84 years) and male to female ratio 0.75 (83:110). Travel was intended to 80 countries for a median of 16 days (range 2 to 3167), predominantly for leisure (53%), non-medical work (17%) and visiting friends and relatives (12%). Live vaccine safety dominated discussion in the pre-travel consultation. Existing guidelines arguably fell short in dealing with travel risks associated with hyper-specific conditions, targeted immunosuppressants and non-vaccine preventable infections.

**Conclusions:**

Our cohort represents a wide spectrum of immunocompromise, for whom we arguably need more measurable ways to approach travel-associated risks. We propose prospective qualitative participatory research to inform our unit of the priorities of ICTs in the pre-travel consultation. We further recommend the formation of a repository of specialists and formulary of complex cases to direct subsequent informative systematic review and prospective risk studies.

**Supplementary Information:**

The online version contains supplementary material available at 10.1186/s40794-024-00217-0.

## Introduction

There were 93.1 million visits overseas by UK residents in 2019, which reflects the 20 year steady increase in overseas travel, prior to the covid-19 pandemic [1]. The expansion of novel therapeutics has brought an increase in immunocompromised travellers (ICTs), and the challenges of managing associated travel risks [2, 3].

### Risk of infection

Immunocompromise increases individuals’ risk of acquiring infections, both standard and opportunistic, of these infections progressing to severe disease and reduces their ability to clear infections [2, 4, 5]. Vaccine-preventable endemic infectious diseases constitute important discussion, but travel medicine concerns much broader risk considerations than ‘travel vaccinations’ alone. Repeat childhood immunisations may be necessary in post-haemopoietic stem cell transplant (HSCT) or post-rituximab ICTs [4]. There are increased risks associated with non-vaccine preventable infections. Some ICTs are at greater risk of developing infections such as tuberculosis, or infections from environmental pathogens such as dimorphic fungi and nontuberculous mycobacteria. Asplenic or hyposplenic individuals have increased susceptibility to infections caused by encapsulated organisms such as pneumococcus, meningococcus and haemophilus as well as more fastidious organisms such as capnocytophagia and babesia [6]. ICTs in general are at greater risk of malaria progressing to severe disease if contracted. Guidance on antimicrobial prophylaxis across immunocompromised groups can be inconsistent. Whilst there is clear evidence for individuals post-HSCT or people living with human immunodeficiency virus (HIV) infection who have a CD4 count < 200 to be on routine prophylactic antimicrobial therapy, there is a lack of evidence in other conditions, such as functional hyposplenia secondary to coeliac disease, or in individuals on biologic therapy. Travel to areas with high rates of antimicrobial resistance combined with an often above average nosocomial exposure and prolonged infections, increases the risk of disease caused by multidrug-resistant organisms [7–9].

#### Vaccine safety and efficacy

The risk of live vaccine-associated disease must be balanced against the risk of vaccine-preventable infectious disease [10, 11]. Compromised immune systems may result in limited response to inactivated travel vaccines such as rabies, hepatitis A or typhoid [12, 13]. There remains a paucity of evidence around dosing of vaccines in immunocompromised individuals. For example whilst there is evidence to support double dosing of Hepatitis B vaccination in some conditions including HIV and post-transplantation, this remains under investigated in other immunocompromised groups [14–16]. Frequently, pre-travel serology is required to monitor antibody response, and in the context of rituximab or HSCT, vaccination may need to be deferred until 3–24 months after treatment completion [17]. In some specific situations such as in patients following thymectomy, yellow fever vaccination (YFV) is absolutely contraindicated, but in others such as asplenia, live vaccines do not pose a concern.

### Logistical challenges

The risk of experiencing flares or relapse of underlying medical conditions means ICTs may need to consider the availability of specialist medical care in the travel destination. Trip adjustments may be needed to ensure regular medicine supply, cold chain maintenance and medication timing across time zones. The psychological impact of challenging travel or preventing travel also needs consideration. Road traffic accidents or injuries remain a significant risk to all travellers, and immunocompromise adds to the risk of acquisition of hospital acquired infections and environmental exposure at the time of the trauma.

### Guidelines

Whilst some guidelines exist, there remains a dearth of evidence and literature in this area [18–20]. The dilemma is that there aren’t widely agreed definitions for immunocompetence nor clinical or laboratory correlates for immunocompromise. The matrix of inter-traveller variability makes data gathering and conducting randomised control trials in this group extremely difficult. In our retrospective review, we consider primary and acquired immunocompromise, including chronic conditions such as kidney and liver disease, and diabetes mellitus, that confer immune dysfunction, as well as physiological states. Infancy encapsulates a period of immune system prematurity whilst aging induces immunosenescence [21, 22]. Pregnancy corresponds to a period of relative immunocompromise, increased susceptibility to infection, and as such special considerations when travelling [22, 23].

### Objectives

This paper describes the spectrum of immunocompromise seen amongst ICTs attending the London Hospital for Tropical Diseases (HTD) travel clinic; a specialist physician and clinical nurse specialist led service. The objectives were: (1) To describe the spectrum of immunocompromise in ICTs attending the clinic, (2) to evaluate the travel advice offered and (3) to stratify ICTs by level of travel risk using existing published guidelines.

## Methods

ICT’s attending the HTD Travel Clinic between 1st April 2019 and 30th April 2020 were identified from the electronic health record system (EPIC) and included in the study if they met any of the following criteria: (a) coded as ‘flu at risk’ (b) coded as eligible for ‘shingles Post-Exposure Prophylaxis’ (c) less than two years old (d) over 60 years old (e) pregnant individuals. The “Flu at risk cohort. V6.0” and the ‘post-exposure shingles prophylaxis’ codes include patients that have underlying immunocompromise [24]. Premature or senescent immunity are not defined by age thresholds. For the purposes of this study, age thresholds were chosen in alignment with YFV guidance and its respective evidence to capture these subgroups of travellers with immune system considerations relevant in the travel clinic. Revaccination for yellow fever is advised in individuals who had their first vaccination aged less than two due to premature immune system, and caution is advised when considering administering YFV to adults aged over 60 [25]. Patient data fed into a University College London Hospital SQL server data warehouse called Caboodle. In Caboodle, patients’ SNOMED (coded) diagnosis at the time of the visit, and clinical details were extracted. Individuals were excluded if they a) were not travelling or b) were not systemically immunocompromised on individual review. Data points were extracted directly by EB and BC for each ICT in the cohort from EPIC via coded answers from electronic travel questionnaires that all travellers attending the clinic fill out, or manually from individual clinic visit electronic documentation. The UK Green Book, Center for Disease Control (CDC) Yellow Book, The Infectious Diseases Society of America (IDSA) Guidelines, and shingles Prophylaxis guidance were used to grade severity of immunocompromise.

## Results

**Fig. 1:**
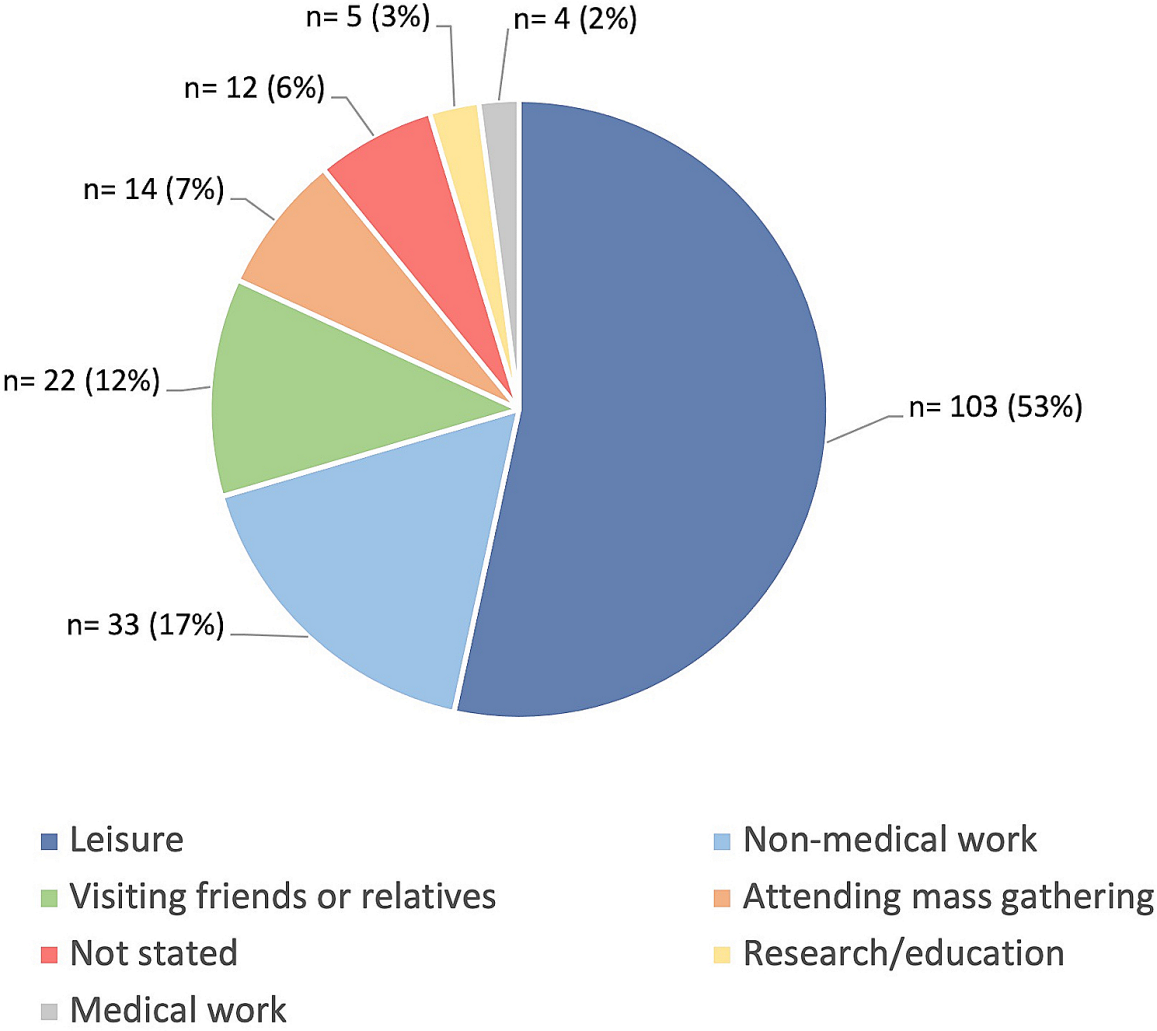
Distribution of primary reason for travel

Out of a total of 1215 travellers who attended the HTD travel clinic between 1st April 2019 and 30th April 2020, 218 potentially immunocompromised travellers were identified. Of these, 25 patients were excluded; 13 were immunocompromised but were not travelling (seven attended for a pneumococcal vaccination; one for a re-issue of a yellow fever certificate, and five for annual or post-deployment medical). Eleven were immunocompetent, inaccurately captured owing to non-systemic immunosuppressants and one traveller’s details could not be retrieved. A total of 193 travellers were included. The ratio of male to female was 0.75 (83/110); median age; 38 (range 9 months to 84 years). ICTs planned trips to 80 different countries. The top 10 most visited countries were Brazil (*n* = 22), Kenya (*n* = 19), Ghana (*n* = 17), Peru (*n* = 13), Tanzania (*n* = 13), Thailand (*n* = 12), India (*n* = 11), Argentina (*n* = 11), South Africa (*n* = 9), and Nigeria (*n* = 9). Generally, ICTs in our clinic were travelling to East and West Africa (19% and 18% respectively), South America (16%), South Eastern Asia (11%) and Southern Asia (8%). Around one third (*n* = 62, 32.6%) ICTs planned to visit more than one country. Travel duration ranged considerably, from 2 to 3167 days, with a median duration of 16 days. Figure 1 outlines the primary reasons for travel among ICTs. (See supplementary index for detailed summary of demographics by reasons for travel).

### Types of Immunocompromise

The distribution of IC is shown in Table 1. Most ICTs had secondary immunocompromise (97.9%) and amongst these the most common reason was physiological (42%), followed by chronic infectious inflammatory conditions (17.1%). Immunocompromise due to treatment with immunomodulating and suppressive medications accounted for 16.6%. A tiny minority (2.1%) had primary immunodeficiency.

**Table 1:** Types of immunocompromise across the immunocompromised traveller cohort

Immunosuppressive Condition or State			Patients (n)		
Primary immunodeficiencies					4
Secondary/Acquired immunocompromise					189
	a. Physiological			81	
		Pregnancy	36		
		Age ≥ 60	34		
		Age ≤ 2	11		
	b. Infectious inflammatory condition			33	
		Living with HIV	31		
		Infectious Liver Disease	2		
	c. Immunosuppression for the following conditions			32	
		Rheumatological	17		
		Neurological	5		
		Gastrointestinal	3		
		Ophthalmological	3		
		SOT	2		
		Dermatological	1		
		PFAPA Syndrome	1		
	d. Malignancy			21	
		Solid organ cancer	1		
		Haematological cancer	20		
	e. Metabolic inflammatory condition			13	
		Diabetes mellitus I & II	13		
	f. Asplenia/hyposplenia			7	
		Sickle cell disease	6		
		Coeliac disease	1		
	g. Thymic dysfunction			2	
		Thymectomy secondary to myasthenia gravis	2		
Total					193

Abbreviations: HIV, human immunodeficiency virus; PFAPA, Periodic Fever, Aphthous Stomatitis, Pharyngitis, Adenitis; SOT, solid organ transplant. Categorisation of ICTs into the above groups reflects the most immunocompromising diagnosis or main immunosuppressive therapy responsible for immunosuppression

Travellers were taking a wide variety of immunosuppressive medications (Table 2). Fifty seven ICTs were taking as many as 23 different immunosuppressive drugs including nine ICTs who were taking two different medications and 1 ICT who was taking three different medications. Five patients were either taking steroids as part of multi-drug regimen, or as a single immunosuppressive agent at a moderate to high dose [18].

**Table 2:** Immunosuppressive therapies across the immunocompromised traveller cohort

Immunosuppressive class and drug			ICTs (n)
Antimetabolite			22
	Azathioprine	8	
	Hydroxycarbamide	3	
	Leflunomide	1	
	Mercaptopurine	1	
	Methotrexate	9	
TNF Inhibitor			7
	Adalimumab	5	
	Etanercept	2	
Steroids			5
Anti-CD20			4
	Rituximab	4	
Mycophenolate mofetil			4
Calcineurin Inhibitor			4
	Sirolimus	1	
	Tacrolimus	3	
α4-integrin inhibitor			3
	Natalizumab	2	
	Vedolizumab	1	
Interferon therapy			2
	Peginterferon alfa-2a	1	
	Interferon beta-1α	1	
BCR-ABL tyrosine kinase inhibitor			1
	Nilotinib	1	
Sphingosine 1-phosphate receptor modulator			1
Anti-IL6			1
	Tocilizumab	1	
CD30 Targeted Agent			1
	Brentuximab	1	
Co-stimulation modulator			1
	Abatacept	1	
IL-12, IL-23 Pathway Inhibitor			1
	Ustekinumab	1	
Total			57

Abbreviations: TNF, tumour necrosis factor; IL, interleukin

### Degree of Immunocompromise

Just three international travel medicine guidelines (Green Book, IDSA, CDC) categorise ‘severity’ of immunocompromise [7, 18, 19, 24, 26]. Stratification is based on live vaccine risk, and does not at present, incorporate risk of acquiring opportunistic infections or condition-related complications. Due to this limitation, we stratified our cohort on this basis. (Tables 3 and 4). The mild ICT group may be considered eligible to receive live vaccination with qualifications, whilst the cohort of severely ICT will almost never be given live vaccines.

**Table 3:** Mildly immunocompromised travellers

Severity	Reason for Immunocompromise	Core condition/state		Immunosuppressive medication or treatment	Patients (n)	
Mild (*n* = 145)	Physiological	Pregnancy [23, 47, 48]		NA	36	81
		Age ≥ 60 [49, 50]			34	
		Age ≤ 2 [22]			11	
	Chronic Infectious Condition	HIV [19]	CD4 ≥ 500mm^3^	NA	26	33
			CD4 ≥ 200 < 500 mm [3]		5	
		Infectious liver disease	Cirrhosis, chronic hepatitis B	NA	1	
			Partial liver secondary to parasitic cyst removal, disseminated candida		1	
	Chronic metabolic condition	Diabetes mellitus [22]		NA	13	13
	Malignancy	Solid cancer	Neuroendocrine tumour, with liver metastases	Radiotherapy < 12 months ago (localised radiotherapy) *	1	1
	Immune modulated conditions on low dose immunosuppression	Bilateral uveitis		Low dose prednisolone < 20 mg/day [18]	1	10
		Psoriasis		Low dose oral methotrexate < 25 mg weekly [18]	1	
		Rheumatoid arthritis			2	
		Systemic lupus erythematous			1	
		Crohn’s disease		Low dose azathioprine < 3 mg/Kg/day [18]	1	
		PFAPA syndrome			1	
		Birdshot uveitis		Low dose MMF ≤ 1 g a day [18]	1	
		Dermatomyositis		azathioprine < 3 mg/Kg/day and prednisolone < 20 mg/day	1	
		Multiple sclerosis [7]		Interferon beta-1α injections	1	
	Hyposplenia	Sickle cell disease [7]		Hydroxycarbamide	3	7
				No treatment	3	
		Coeliac disease		NA	1	

Abbreviations: HIV, human immunodeficiency virus; MMF, mycophenolate mofetil; NA, not applicable; PFAPA, Periodic Fever, Aphthous Stomatitis, Pharyngitis, Adenitis. *CDC suggests severe IC only if “recent”, Green book suggests severe if radiotherapy < 6 months ago therefore we categorised as mild

**Table 4:** Severely immunocompromised travellers

Severity	Reason for Immunocompromise	Core condition/state		Immunosuppressive medication or treatment	Patients (n)	
Severe (*N* = 48)	Primary Immunodeficiency [19]	Combined Variable Immune Deficiency		NA	1	4
		CD4/CD8 idiopathic lymphocytopenia			1	
		Undefined primary immune deficiency			1	
		CD4 T lymphocyte deficiency			1	
	Malignancy	Haematological Cancer	Myeloproliferative Neoplasm: POEMS Syndrome (Castleman Variant)	HSCT < 24 months [7]	1	20
			Non-Hodgkin’s Lymphoma: High grade relapsed follicular lymphoma		1	
			Non-Hodgkin’s Lymphoma: Diffuse large B-cell lymphoma		2	
			Hodgkin’s Lymphoma		2	
			ALL (Acute lymphoblastic leukaemia)		1	
			Non-Hodgkin’s Lymphoma	HSCT > 24 months plus GVHD [18]	1	
			ALL (acute lymphoblastic leukaemia)		1	
			ALL (Acute lymphoid leukaemia)	< 24 months after CAR-T therapy, IT MTX low dose [7]	1	
			Non-Hodgkin’s (Follicular lymphoma)	Rituximab (Anti CD20) [18, 19]	3	
			Non-Hodgkin’s (MALT-lymphoma)		1	
			Non-Hodgkin’s lymphoma	IT MTX (low dose) [18]	1	
			Chronic lymphoproliferative disorder: Chronic Myeloid Leukaemia [18]	Nilotinib (BCR-ABL tyrosine kinase inhibitor) [7, 51]	1	
			Chronic Lymphoproliferative disorder: CLL (chronic lymphocytic leukaemia) [18]	NA	1	
			Non-Hodgkin’s: T-Lymphoblastic Lymphoma	Maintenance vincristine, methotrexate > 25 mg weekly, mercaptopurine > 1.5 mg/Kg/day [26]	1	
			Myeloproliferative disease: Waldenström macroglobulinaemia [18]	NA	1	
			Myeloproliferative neoplasm Polycythaemia rubra vera	Peginterferon alfa-2a [52]	1	
	Immune Modulated Conditions on significant dose immunosuppression	SOT	Renal transplant	Tacrolimus, sirolimus [7]	2	22
		Rheumatological Conditions	Rheumatoid arthritis	TNF inhibitors [19]	2	
			Juvenile arthritis		3	
			Sacroiliitis		1	
			Enthesitis related arthritis/uveitis		1	
			Rheumatoid arthritis	Rituximab < 6 months [19]	1	
			Primary Sjogren’s syndrome	MMF > 1 g a day [7]	1	
			SRP-positive polymyositis	MMF/calcineurin inhibitors/steroids [7]	1	
			Juvenile Arthritis	Co-stimulation modulator Abatacept [51]	1	
			Rheumatoid arthritis	Anti-IL-6 agent tocilizumab [7]	1	
			Psoriatic arthritis	High dose steroids [18, 19]	1	
		Neurological	Multiple sclerosis		1	
				α4-integrin inhibitor natalizumab [7, 53]	2	
				Fingolimod [7]	1	
		Gastroenterological	Crohn’s disease	IL-12,IL-23 pathway inhibitor ustekinumab [7, 53]	1	
				Vedolizumab, azathioprine < 3 mg/Kg/day [53, 54]	1	
		Ophthalmological	Chorioretinitis (clinically active)	MMF > 1 g/day [7]	1	
	Thymic Dysfunction	Myasthenia Gravis		Thymectomy [55]	2	2

Abbreviations: CAR-T Therapy, chimeric antigen receptor T-cell therapy; GVHD, graft versus host disease; IT, intrathecal; IL, interleukin, HCST, haematopoietic stem cell transplant; MTX methotrexate; MMF, mycophenolate mofetil; NA, not applicable

Table S5 (Supplementary Index) compares the grading categories across the guidelines. CDC, IDSA and the Green Book (UK) largely align, but we found that the Green Book and IDSA do not provide detail to differentiate risk between different individual biologics, and whilst most conditions are grouped, some are not directly mentioned. The vaccine risk profile of a tyrosine kinase inhibitor such as nilotinib, being taken by one of the ICTs in our study, is not discussed specifically in any of these 3 published vaccine safety guidelines. Myeloproliferative diseases such as polycythaemia rubra vera are not highlighted. We have categorised this individual as severe due to having a haematological neoplastic disorder. Some discrepancies exist between sources in the definition of risk period following solid organ transplantation (SOT) and chemoradiotherapy. There is some ambiguity in the context of multiple sclerosis; CDC guidance highlights interferon as a therapeutic agent that confers severe immunocompromise but notes it is considered immunomodulation by specialists and therefore would not be contraindicated in live vaccination. The definitions of ‘high’ dose immunomodulation for individuals taking azathioprine, methotrexate, mercaptopurine and corticosteroids align across all sources [18].

In this study, no severely ICT at time of appointment, according to our list, were given live vaccines. We have highlighted conditions that the guidelines do not cover and additional study subgroups in grey.

### Travel advice

Out of 193 ICTs, 2.1% (*n* = 4) were advised against travel. Half (47.7%, *n* = 92) of all ICTs were travelling to malaria endemic areas and were issued malaria chemoprophylaxis. Of these ICTs, 39.1% (*n* = 36) were ‘special category’ individuals requiring second-line prophylaxis due to pregnancy, breastfeeding, age < 2 years, and potential drug-to-drug interactions [27].

About a fifth of ICTs (18.7%, *n* = 36) had serology testing. The most frequently offered vaccination was hepatitis A (*n* = 49), rabies (*n* = 40) typhoid (*n* = 34), pneumococcal (*n* = 34), diphtheria tetanus and polio (*n* = 33), yellow fever (*n* = 21), meningitis ACWY (*n* = 12), hepatitis B (*n* = 12), and measles mumps rubella (MMR) (*n* = 7). MMR and YFV was contraindicated in five and 28 ICTs respectively due to severe immunocompromise at time of appointment. Additional vaccinations such as Japanese encephalitis virus (*n* = 8), tetanus (*n* = 6), meningitis B (*n* = 3), Haemophilus influenzae B (*n* = 3), human papilloma virus (*n* = 2), cholera (*n* = 2), meningitis C (*n* = 2) were given to a small fraction. Discussion surrounding rabies or pneumococcal vaccine was not consistently documented to understand the extent to which these were considered across the cohort. Additional individualised considerations included issuing medic-alert bracelets, discussion about risk of tuberculosis exposure, and the logistics of maintaining a cold chain for transporting medicines were relevant for 13.5% (*n* = 26) of all ICTs. Just 10.4% (*n* = 20) of all travellers were issued rescue pack antibiotics (immunomodulated inflammatory rheumatological conditions *n* = 7, haematological malignancy *n* = 5, multiple sclerosis *n* = 2, HIV *n* = 2, diabetes *n* = 1, solid malignancy *n* = 1, SOT *n* = 1, primary immunodeficiency *n* = 1).

### Severe immunocompromise

Of the 48 severely ICTs, 6.25% (*n* = 3) were advised not to travel. The first was an individual with a primary immunodeficiency travelling to India for a non-medical work trip. The second individual was significantly immunosuppressed with a TNF inhibitor for juvenile arthritis, primary immunodeficiency, and neurological co-morbidities, travelling to Pakistan for a mass gathering. The third traveller had clinically active autoimmune eye disease who had just commenced mycophenolate, travelling to Vietnam for leisure.

Live vaccine safety dominated the documented clinical discussion and reasons for attendance. This included discussing the timing of (re-vaccination schedule after haemopoietic stem cell transplant or biologic therapy, serology testing or YFV exemption or vaccine contraindication discussion.

YFV was contraindicated for all 10 travellers visiting yellow fever endemic areas. These travellers were exempt due to history of thymectomy (*n* = 1), taking a TNF inhibitor (*n* = 1), natalizumab (*n* = 2), HCST within 24 months (*n* = 1), taking ustekinumab (*n* = 1 where timing of yellow fever was discussed for three months after they stop taking this monoclonal agent), recent high dose steroid courses (*n* = 1, YFV was discussed after disease had stabilized), cladribine (*n* = 1), and high dose mycophenolate (*n* = 1).

MMR vaccination was contraindicated in five severely ICTs travelling to Australia, Canada, Colombia, Sri Lanka, United Arab Emirates and Thailand. Three had a haematological malignancy; one was < 2 years post-HSCT, one was < 2 years post- chimeric antigen receptor T-cell (CAR-T) therapy and one was < 6 months post rituximab treatment. One was taking a TNF inhibitor and one had primary immunodeficiency post-HCST without full immune reconstitution. Two of these ICTs had no residual measles immunity (“IgG negative”). One was able to have an MMR prior to departure, as this was beyond the two-year mark, and the other ICT was advised they could travel but to avoid mass gatherings. The remaining three had residual measles antibodies on serology. The ICT six months-post rituximab was advised with small delay he could receive MMR prior to travelling to Colombia.

A fifth had additional discussions around cold chain management in the context of transporting insulin, and adalimumab. Approximately a third received antibiotic rescue packs.

### Mild Immunocompromise

Of the 145 mildly ICTs, one pregnant individual travelling to Uganda for non-medical work was advised against travelling. The YFV was contraindicated or exempt for just under half (46.2%, *n* = 18) of the 39 mildly IC travellers visiting areas with potential yellow fever risk. Exceptions were given based on age < 2, or > 60 with or without additional co-morbidities, clinically active eye disease, pregnancy, and an individual living with HIV with a high viral load. Of the 21 travellers who received the YFV, individuals were 60 or over (*n* = 4); without co-morbidities (*n* = 2), and with co-morbidities such as coeliac or diabetes (*n* = 2), individuals between 6 months and 2 years old without underlying health conditions (*n* = 2), or with sickle cell disease (*n* = 1), aged 18–59 living with virologically supressed HIV CD4 > 200 (*n* = 7), sickle cell disease (*n* = 3), diabetes (*n* = 2) or on low dose antimetabolites (*n* = 2).

The MMR vaccine was offered to seven individuals in the mild group. This was as a first scheduled MMR dose in children less than two years old without other conditions as per childhood immunization schedule, and second dose in two individuals less than six years old with sickle cell disease. Three individuals living with well controlled HIV, diabetes, and one who was pregnant at the time were offered MMR at appropriate timings due a history of incomplete MMR vaccination. Thirty-four ICTs were relatively immunocompromised due to being aged 60 and over. In this group, YFV decisions were made on a case-by-case basis. The pneumococcal vaccine and influenza vaccine status of the traveller was reviewed in those 65 and over. Non-immunocompromising co-morbidities were frequent in this group, with 26 out of 34 (76.5%) taking routine medications (e.g. statins, antihypertensives, inhaled salbutamol). Discussion specifically included management of co-morbidities during travel, travel insurance, and ensuring access to medical professional advice if relevant. Just 3.5% (*n* = 5) mildly ICTs were issued rescue antibiotic packs.

## Discussion

There was a wide spectrum of hyper specific immunocompromise across the cohort attending HTD travel clinic during this period, with wide inter-traveller variability in destination, duration, and reason for travel. Around half of all individuals were travelling to malaria-endemic areas and a quarter to yellow fever endemic areas. This may reflect a widespread perception that travel associated risks mostly pertain to travel-vaccine preventable diseases and malaria chemoprophylaxis. The focus of documented discussion on live vaccination may reflect the practitioner’s dilemma of balancing the risk of iatrogenic harm administering a live vaccine, with the risk of withholding a vaccine for an at-risk area. Current travel medicine guidelines focus on live vaccines by limiting stratification of ICTs into ‘high’ and ‘low’ risk of adverse events from live vaccination administration. Even in stratification, broad categories of ICTs are stated, but there is a lack of nuance to guide practitioners about risks associated with specific individual conditions, targeted biologics, and novel immunosuppressants. Furthermore, with the rapid development of new drugs and drug classes, the guidelines for advising ICTs is continuously behind.

### Live vaccination safety

Yellow fever and MMR were the live vaccines most relevant to our cohort. Yellow fever is a relatively avoidable, rare condition to affect travellers. There has only been a total of 32 reported yellow fever cases and four known fatalities in unvaccinated international travellers [28–30]. With this in mind, the risk of YFV associated neurological disease (YEL-AND) is estimated at 2.2 cases per 100,000 doses of vaccine in individuals over 60, and 0.8 cases per 100,000 doses in individuals under 60, and carries a 2% case fatality rate [31]. The risk of YFV associated viscerotropic disease (YEL-AVD) is estimated at 1.2 cases per 100,000 doses in over 60’s, and 0.3 cases per 100,000 doses in under 60s and carries a 48% fatality rate [31]. Our threshold for replacing vaccination with bite prevention was and is low. Small studies suggest YFV is safe in some ICT groups e.g., ICTs taking natalizumab, infliximab, indicating there is further work to be done on individual target specific agents to reach consensus opinion [32–37].

Measles outbreaks remain a global issue including in the UK, with severe consequences, therefore the emphasis is to support safe vaccination wherever possible [38–40]. Severe complication of live MMR vaccine is a rare occurrence. There have been 66 published laboratory-cases of vaccine-derived measles to date internationally, with three fatalities occurring in individuals with primary immunodeficiency disorders [41]. Small studies suggest MMR vaccination is safe > 2 years post-HSCT [42–45]. There was one reported case of vaccine-derived measles in an individual two months-post graft versus host disease resolution, and three years post-HCST; they experienced a benign self-limiting rash [41]. There has been one case of vaccine-associated measles in an individual who was taking natalizumab, with full recovery [46]. Natalizumab specifically prevents lymphocyte migration into the central nervous system therefore there is a theoretical enhanced risk of vaccine derived subacute sclerosing panencephalitis, but there have been no reported cases to date.

### Recommendations

Prospective participatory qualitative research will be essential in understanding the main priorities, concerns and expectations of ICTs when travelling compared to those of advising health professionals. Follow up post travel is important to capture how likely individuals are to make behavioural adjustments if advised, and the incidence of health-related complications during travel. Given the heterogeneity of ICTs and continuous emergence of novel immunosuppressants it is extremely difficult to build a straightforward guideline with the combined lack of evidence supporting individual vaccine safety decisions and variable epidemiological factors influencing risk of acquiring infection, such as seasonality, human behaviours, and duration of travel. However, we arguably need more measurable ways to look at risk, to provide clearer evidence-based guidance and interventions, to allow ICTs to make informed decisions, and consider wider infection prevention and control implications. Binary categorisation of risk does not currently map the need for individualised medicine. A radar graph or sliding scale approach across different risk domains could be one approach to take. A formulary of complex cases is one suggestion we put forward in the first instance, and the creation of a repository of specialists to provide expert opinion on case management, and on the direction of prospective research into risk domains of immunocompromised travel. There is a need for formal systematic review of the evidence base underpinning existing current guidelines. This would help to map the gaps in evidence, to shape an agenda for prospective work.

### Electronic supplementary material

Below is the link to the electronic supplementary material.

Supplementary Material 1

## Data Availability

No datasets were generated or analysed during the current study.
