# Supplementary material for: Advising the immunocompromised traveller: a review of immunocompromise at The London Hospital for Tropical Diseases Travel Clinic between 1st April 2019 and 30th April 2020
Source: Trop Dis Travel Med Vaccines. 2024 Apr 15;10:8. doi: 10.1186/s40794-024-00217-0 (PMC11017494; doi:10.1186/s40794-024-00217-0)
Supplement: Supplementary file 1 — Supplementary Material 1 [file 40794_2024_217_MOESM1_ESM.pdf]

Supplementary Index

Table S1

Demographics and Travel Characteristics of 193 patients attended HTD Travel Clinic, according to reason of travel, 1st April 2019 – 30th April 2020

		All Travellers n (%)	Leisure n (%)	VFR n (%)	Mass Gathering n (%)	Medical Work n (%)	Non-medical Work n (%)	Research n (%)
**Gender**	**Male**	83 (43.0)	56 (45.2)	12 (40.0)	5 (35.7)	1 (25.0)	16 (48.5)	2 (25.0)
	**Female**	110 (57.0)	68 (54.8)	18 (60.0)	9 (64.3)	3 (75.0)	17 (3.1)	6 (75.0)
**Age**	**<2**	13 (6.7)	10 (8.1)	2 (10.0)	0	0	2 (6.1)	0
	**03-17**	9 (4.7)	5 (4.0)	1 (5.0)	0	0	3 (9.1)	0
	**18-29**	24 (12.4)	18 (14.5)	1 (5.0)	3 (21.4)	1 (25.0)	2 (6.1)	2 (25.0)
	**30-59**	85 (44.0)	48 (38.7)	14 (70.0)	5 (35.7)	2 (50.0)	17 (51.5)	5 (63.0)
	**> 60**	62 (31.1)	43 (34.7)	2 (10.0)	6 (42.9)	1 (25.0)	9 (27.3)	1 (13.0)
**Severity**	***Mild***	*145 (75.1)*	*83 (66.9)*	*24 (80.0)*	*9 (64.3)*	*4 (100.0)*	*28 (84.8)*	*7 (87.5)*
	Severe	*48 (24.9)*	*41 (33.1)*	*6 (20.0)*	*5 (35.7)*	*0 (0.0)*	*5 (15.2)*	*1 (12.5)*
**Travelled Regions**	**Australia and New Zealand**	2 (1.04)	1 (0.81)	1 (3.33)	0	0	1 (3.03)	0
	**Caribbean**	6 (3.11)	6 (4.84)	0	0	0	0	0
	**Central America**	4 (2.07)	3 (2.42)	0	0	0	0	0
	**Eastern Africa**	43 (22.3)	26 (21.0)	6 (20.0)	3 (21.4)	2 (50.0)	8 (24.2)	3 (37.5)
	**Eastern Asia**	5 (2.6)	3 (2.42)	1 (3.33)	0	0	2 (6.06)	0
	**Eastern Europe**	4 (2.07)	2 (1.61)	0	0	0	2 (6.06)	0
	**Melanesia**	1 (0.52)	0	0	0	0	1 (3.03)	0
	**Middle Africa**	1 (0.52)	1 (0.81)	0	1 (7.1)	0	0	0
	**Northern Africa**	2 (1.04)	1 (0.81)	0	0	0	0	0
	**Northern America**	2 (1.04)	2 (1.61)	0	0	0	0	1 (12.5)

	**South America**	33 (17.1)	24 (19.4)	0	0	0	3 (9.09)	1 (12.5)
	**South Eastern Asia**	23 (11.9)	22 (17.7)	6 (20.0)	0	0	2 (6.06)	0
	**Southern Africa**	11 (5.70)	9 (7.26)	1 (3.33)	1 (7.14)	1 (25.0)	1 (3.03)	1 (12.5)
	**Southern Asia**	15 (7.77)	8 (6.45)	5 (16.7)	1 (7.14)	0	2 (6.06)	1 (12.5)
	**Western Africa**	35 (18.13)	13 (10.5)	10 (33.3)	6 (42.9)	0	11 (33.3)	1 (12.5)
	**Western Asia**	6 (3.11)	3 (2.42)	0	2 (14.3)	1 (25.0)	0	0
**Travelled Countries (top 10)**		Brazil 16 (8.3)	Kenya 10 (8.1)	Brazil 6 (20)	Ghana 3 (21.4)	Zambia 1 (25.0)	Nigeria 3 (9.1)	Malawi 2 (25.0)
		Ghana 16 (8.3)	Brazil 10 (8.1)	Ghana 4 (13.3)	Saudi Arabia 2 (14.3)	Yemen 1 (25.0)	Sierra Leone 3 (9.1)	Nepal 1 (12.5)
		Kenya 14 (7.3)	Thailand 9 (7.3)	Ethiopia 3 (10.0)	Niger 2 (14.3)	Ethiopia 1 (25.0)	Uganda 3 (9.1)	United States 1 (12.5)
		Thailand 9 (4.7)	Peru 8 (6.5)	India 3 (10.0)	Ethiopia 1 (7.1)	South Africa 1 (25.0)	Tanzania 2 (6.1)	South Africa 1 (12.5)
		India 9 (4.7)	Ghana 8 (6.5)	Pakistan 2 (6.7)	Tanzania 1 (7.1)		Ghana 2 (6.1)	Ghana 1 (12.5)
		South Africa 8 (4.1)	South Africa 6 (4.8)	Nigeria 2 (6.7)	South Africa 1 (7.1)		Kenya 2 (6.1)	Ethiopia 1 (12.5)
		Peru 8 (4.1)	Uganda 5 (4.0)	Kenya 2 (6.7)	Uganda 1 (7.1)		Liberia 2 (6.1)	Chile 1 (12.5)
		Ethiopia 8 (4.1)	Ethiopia 4 (3.2)	Niger 2 (6.7)	Pakistan 1 (7.1)			
		Uganda 8 (4.1)	Indonesia 4 (3.2)		Angola 1 (7.1)			
		Nigeria 6 (3.1)	India 4 (3.2)		Nigeria 1 (7.1)			
**Number of countries per travel**	**1 Country**	193 (100)	124 (100)	30 (100)	14 (100)	4 (100)	33 (100)	8 (100)
	**>1 Country**	62 (32.1)	43 (34.7)	5 (16.7)	4 (28.6)	0	11 (33.3)	3 (37.5)
	**Median**	16	15	16	23	33	18	14
	**Mean**	79	67	26	35	43	145	17

**Duration of Travel (In Days)**	**IQR**	12	9	7	14	44	24	14
	**Minimum**	2	2	10	10	14	4	7
	**Maximum**	3167	3167	121	116	92	1490	32
**Reason for Travel**	**Attending mass gathering**	7						
	**Leisure**	124						
	**VRF**	30						
	**Non-Medical Work**	45						
	**Medical Work**	6						
	**Research/Education**	8						
	**Not Stated**	12						

Table S2

Travellers, n, by Geo-region and Country*

**South America**	**64**
Brazil	22
Peru	13
Argentina	11
Chile	6
Colombia	5
Bolivia	4
Ecuador	2
Uruguay	1
**Eastern Africa**	**57**
Kenya	19
Tanzania	13
Uganda	8
Ethiopia	7
Zambia	4
Malawi	3
Mozambique	1
Rwanda	1
Zimbabwe	1
**Western Africa**	**45**
Ghana	17
Nigeria	9
Sierra Leone	8
Liberia	4
Niger	2
Benin	2
Gambia	1
**Eastern Asia**	**12**
China	6
South Korea	3
Mongolia	2
Japan	1
**Central America**	**9**
Costa Rica	3
Panama	3
Mexico*	1
Belize	1
Guatemala	1
**Caribbean**	**8**
Barbados	2
Cayman Islands	1
St Lucia	1
Jamaica	1
Cuba	1
Turks and Caicos	1
Grenada	1
**Western Asia**	**7**
United Arab Emirates	2
Saudi Arabia	2
Yemen	1
Lebanon	1
Israel	1
**Eastern Europe**	**6**
Russia	3

Ivory Coast	1
Senegal	1
**South Eastern Asia**	**42**
Thailand	12
Indonesia	8
Vietnam	7
Cambodia	5
Malaysia	4
Singapore	3
Myanmar	1
Philippines	1
Laos	1
**Southern Asia**	**21**
India	11
Sri Lanka	5
Pakistan	2
Maldives	2
Nepal	1
**Southern Africa**	**14**
South Africa	9
Namibia	4
Botswana	1
Belarus	1
Poland	1
Romania	1
**Australia and New Zealand**	**4**
Australia	3
New Zealand	1
**Northern America**	**3**
United States	2
Canada	1
**Northern Africa**	**2**
Morocco	2
**Middle Africa**	**1**
Angola	1
**Melanesia**	**1**
Papua New Guinea	1
**Polynesia**	**1**
Tonga	1

**2 patients stated they intended to travel to ‘South America’, or ‘South America and Africa’ but had intended to make country decisions following consultation. They were not included in the above table.*

***Included in Central America under the United Nations geo-regions annex.*

Figure S3

<u>Intended destination countries across the immunocompromised traveller cohort</u>

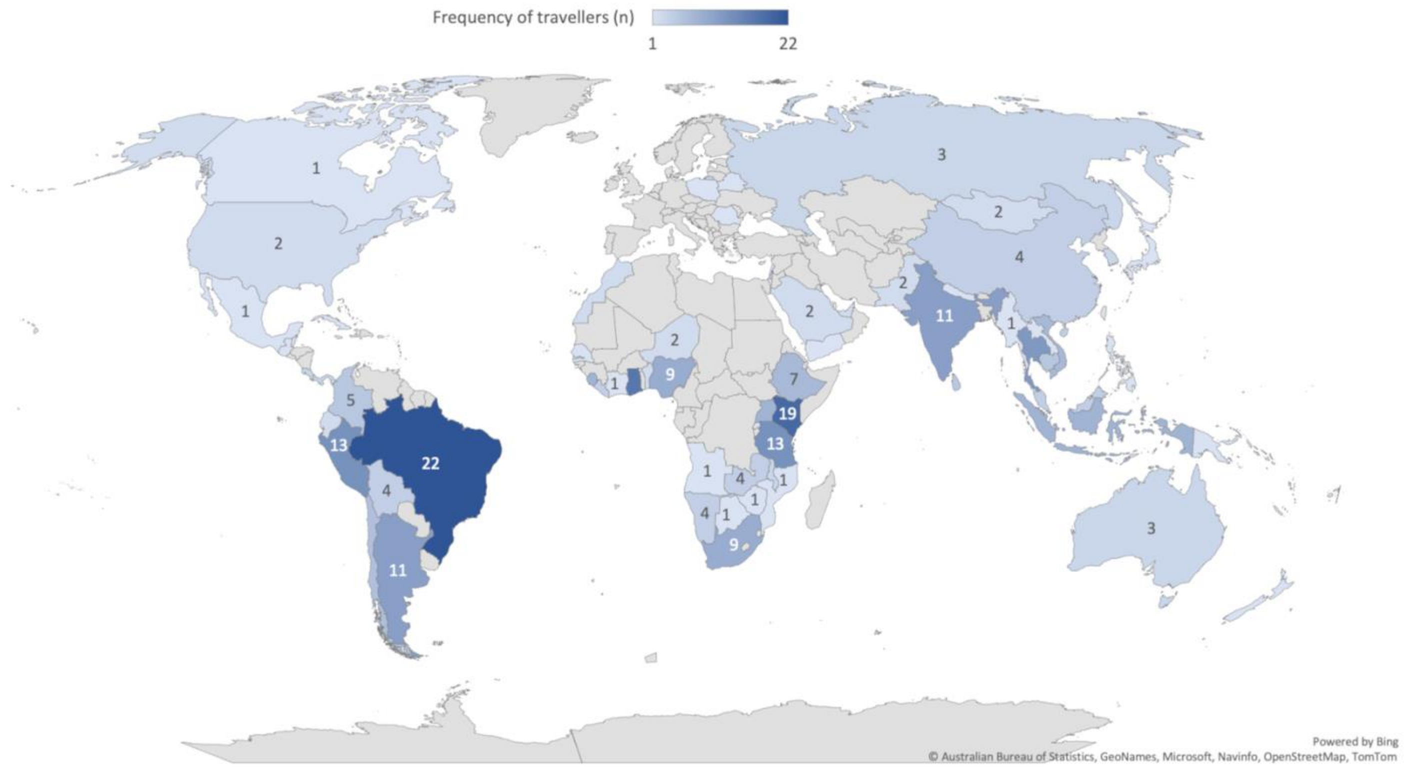

Figure S4

Primary reason for travel by age across the immunocompromised traveller cohort

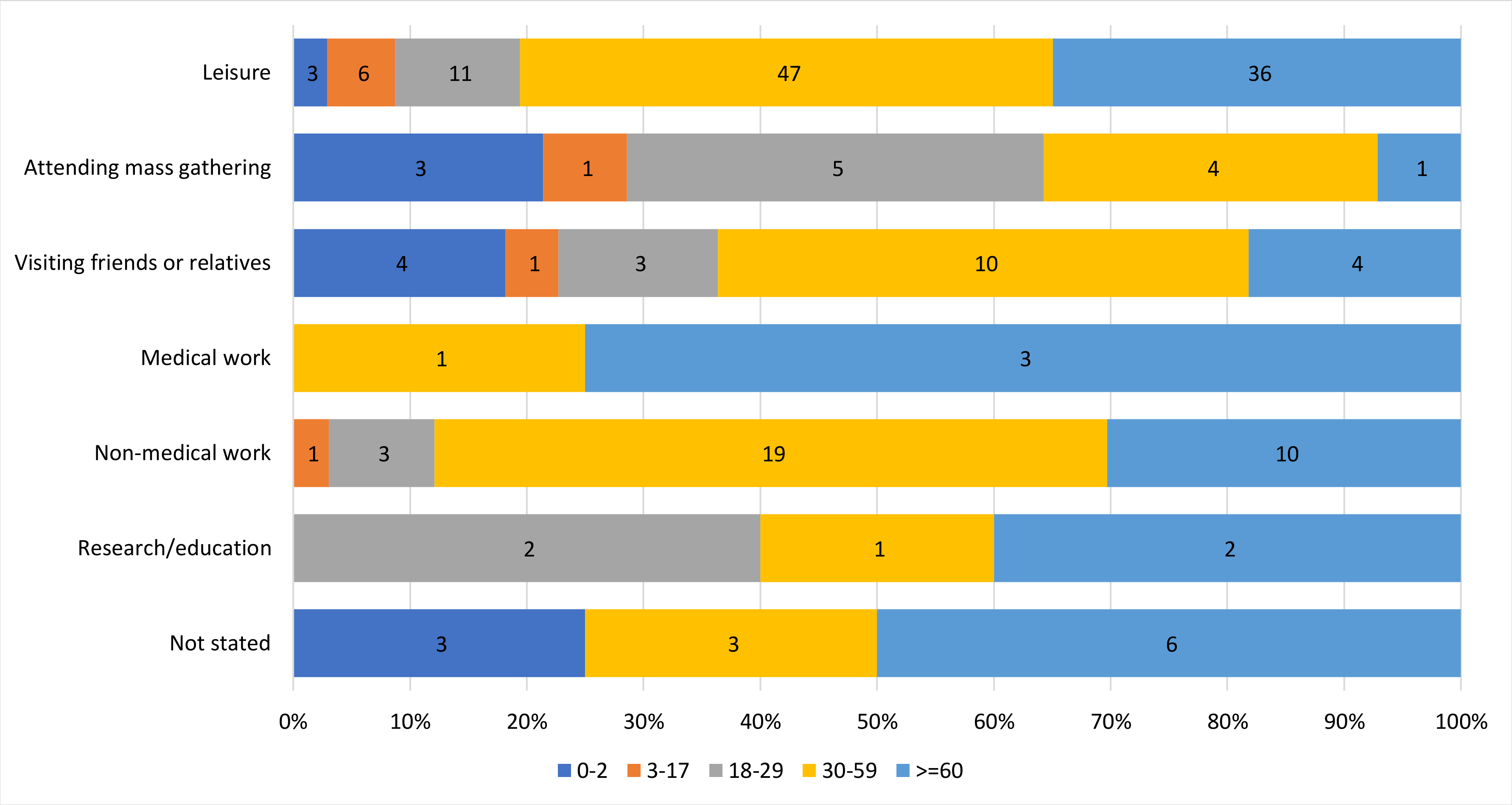

Table S5

Immunosuppression grading and wording across three main sources

Stratification	CDC Yellow Book (1)	IDSA (specific vaccine related advice) (2)	UK Green Book (3)
'No significant immune deficit' (CDC)<br>*'Low level immunosuppression' (IDSA)*	*'Asymptomatic HIV CD4 cells ≥200/ mm3'*<br>*Last chemotherapy >=3 months ago*<br>*Short or long term daily or alternate day prednisolone <20mg equivalent*<br>*>1 month since high dose steroid use*	• *'Asymptomatic HIV CD4 200–499 cells/mm^3*<br>• *Individuals taking lower daily dose of systemic corticosteroid than for high-level immunosuppression for ≥14 days or receiving alternate-day corticosteroid therapy*<br>• *methotrexate (MTX) ≤0.4 mg/kg/week, azathioprine ≤3 mg/kg/day, or 6-mercaptopurine ≤1.5 mg/kg/day'*	Not applicable
'Varying degree of immune deficit' (CDC)	• *Asplenia (e.g. secondary to sickle cell disease)*<br>• *Chronic renal disease*<br>• *Multiple sclerosis*	Not applicable	Not applicable
Significant immune deficit	• *CD4 cells <200/ mm3*<br>• *SOT <1 year*<br>• *congenital immunodeficiency*<br>• *active leukaemia, lymphoma*<br>• *generalised malignancy*<br>• *aplastic anaemia*<br>• *GVHD*<br>• *'recent radiotherapy'*<br>• *CAR-T <2 years*<br>• *HCST <2 years or still on immunosuppressive drugs*	• *HIV CD4 <200 cells/mm^3 for adults and adolescents and percentage <15 for infants and children*<br>• *Within 2 months after SOT*<br>• *combined primary immunodeficiency disorder (eg, severe combined immunodeficiency)*<br>• *receiving cancer chemotherapy*<br>• *receiving daily corticosteroid therapy with a dose ≥20 mg prednisolone or equivalent (or >2 mg/kg/day for patients who weigh <10 kg) of prednisone or equivalent for ≥14 days, and*<br>• *receiving certain biologic immune modulators, that is, a tumor necrosis factor-alpha (TNF-α) blocker or rituximab.*	• *'HIV/AIDS'*<br>• *SOT therapy <6 months (with exceptions, depending upon the type of transplant and the immune status of the patient)*<br>• *cellular immune deficiencies*<br>• *acute and chronic leukaemias and lymphoma*<br>• *under follow up for a chronic lymphoproliferative disorder including haematological malignancies such as indolent lymphoma, chronic lymphoid leukaemia, myeloma and other plasma cell dyscrasias (list not exhaustive)*<br>• *HSCT (allogenic) <2 years or >2 years if they are demonstrated to have on-going immunosuppression or GVHD*<br>• *Autologous HSCT <2 years, or >2 years not in remission*

	• 'High dose corticosteroids >2mg/Kg body weight or ≥20mg /day prednisolone equivalent'<br>• Alkylating agents<br>• Antimetabolites methotrexate >0.4 mg/kg/week, azathioprine >3 mg/kg/day, or 6-mercaptopurine >1.5 mg/kg/day)<br>• SOT medications e.g. cyclosporine, tacrolimus, sirolimus, azathioprine, mycophenolate mofetil<br>• Cancer chemotherapeutic drugs<br>• TNF inhibitors<br>• Other biologics (includes a reference table for immunosuppressants contraindicated with live vaccination)	• After HSCT, duration of high-level immunosuppression is highly variable and depends on type of transplant (longer for allogeneic than for autologous), type of donor and stem cell source, and posttransplant complications such as graft vs host disease (GVHD) and their treatments.	• <6 months immunosuppressive chemotherapy or radiotherapy for malignant disease or non-malignant disorders<br>• immunosuppressive biological therapy <last 6 months (e.g. anti-TNF therapy such as alemtuzumab, ofatumumab and rituximab) unless otherwise directed by a specialist<br>• those who are receiving or have received in the past 3 months immunosuppressive therapy including:<br>- adults and children on high-dose corticosteroids (>40mg prednisolone/day or 2mg/kg/day in children under 20kg) >1 week<br>- adults and children on lower dose corticosteroids (>20mg prednisolone per day or 1mg/kg/day in children under 20kg) >14 days<br>- adults on non-biological oral immune modulating drugs e.g. methotrexate >25mg/week, azathioprine >3.0mg/kg/day or 6-mercaptopurine >1.5mg/kg/day<br>- for children on non-biological oral immune modulating drugs (except those on low doses, see below), specialist advice should be sought prior to vaccination<br>- 'Long term stable low dose corticosteroid therapy, either alone or in combination with low dose non-biological oral immune modulating drugs (e.g. methotrexate 25mg/ week in adults or up to 15mg/m2 in children, azathioprine 3.0mg/ kg/day or 6-mercaptopurine 1.5mg/kg/day), are not considered sufficiently immunosuppressive and these patients can receive live vaccines.'

1. Camille Nelson Kotton ATK, David O. Freedman. Chapter 5: Travelers with Additional Considerations: Immunocompromised Travelers. CDC Yellow Book. New York: Oxford University Press; 2020.
2. Rubin LG, Levin MJ, Ljungman P, Davies EG, Avery R, Tomblyn M, et al. 2013 IDSA Clinical Practice Guideline for Vaccination of the Immunocompromised Host. Clinical Infectious Diseases. 2013;58(3):e44-e100.
3. UK Health Security Agency. Green Book: Immunisation against infectious disease. Public Health England; 2017.
